# Flavanone-3-Hydroxylase Plays an Important Role in the Biosynthesis of Spruce Phenolic Defenses Against Bark Beetles and Their Fungal Associates

**DOI:** 10.3389/fpls.2019.00208

**Published:** 2019-02-25

**Authors:** Almuth Hammerbacher, Dineshkumar Kandasamy, Chhana Ullah, Axel Schmidt, Louwrance P. Wright, Jonathan Gershenzon

**Affiliations:** ^1^Department of Zoology and Entomology, Forestry and Agricultural Biotechnology Institute, University of Pretoria, Pretoria, South Africa; ^2^Department of Biochemistry, Max Planck Institute for Chemical Ecology, Jena, Germany; ^3^Zeiselhof Research Farm, Pretoria, South Africa

**Keywords:** taxifolin, catechin, flavonoids, F3H, *Picea abies*, *Endoconidiophora polonica*, *Ips typographus*

## Abstract

Conifer forests worldwide are becoming increasingly vulnerable to attacks by bark beetles and their fungal associates due to the effects of global warming. Attack by the bark beetle *Ips typographus* and the blue-stain fungus it vectors (*Endoconidiophora polonica*) on Norway spruce (*Picea abies*) is well known to induce increased production of terpene oleoresin and polyphenolic compounds. However, it is not clear whether specific compounds are important in resisting attack. In this study, we observed a significant increase in dihydroflavonol and flavan-3-ol content after inoculating Norway spruce with the bark beetle vectored fungus. A bioassay revealed that the dihydroflavonol taxifolin and the flavan-3-ol catechin negatively affected both *I. typographus* and *E. polonica*. The biosynthesis of flavan-3-ols is well studied in Norway spruce, but little is known about dihydroflavonol formation in this species. A flavanone-3-hydroxylase (F3H) was identified that catalyzed the conversion of eriodictyol to taxifolin and was highly expressed after *E. polonica* infection. Down-regulating F3H gene expression by RNA interference in transgenic Norway spruce resulted in significantly lower levels of both dihydroflavonols and flavan-3-ols. Therefore F3H plays a key role in the biosynthesis of defense compounds in Norway spruce that act against the bark beetle-fungus complex. This enzyme forms a defensive product, taxifolin, which is also a metabolic precursor of another defensive product, catechin, which in turn synergizes the toxicity of taxifolin to the bark beetle associated fungus.

## Introduction

Flavonoids are specialized or secondary plant metabolites that are universally distributed in all vascular plants and occur in many algae as well (Treutter, 2006; Mouradov and Spangenberg, 2014). Containing a C_6_–C_3_–C_6_ backbone of two aromatic rings connected by a C_3_ bridge, they are known to perform numerous internal functions in plants as well as in interactions with the environment. Flavonoids influence the central processes of plant development and nutrition by regulating auxin transport (Kuhn et al., 2011), pollen tube growth (Ylstra et al., 1992) and nitrogen fixation (Ferreyra et al., 2012). They are also involved in negative or positive plant interactions with other organisms. They, for example, inhibit the growth of disease-causing microorganisms (Goetz et al., 1999; Ullah et al., 2017) and deter herbivore feeding (Dübeler et al., 1997; Ohse et al., 2017). On the other hand, these compounds play an important role in attracting pollinators to flowers and dispersal agents to fruits. Flavonoids are well known anti-oxidants that increase plant tolerance to soil salinity (Mahajan and Yadav, 2014), cold stress (Meng et al., 2015) as well as to drought stress (Song et al., 2016). They protect against UV radiation by anti-oxidant effects and direct screening (Ferreyra et al., 2012). The anti-oxidant properties of these molecules are also valuable in human nutrition and contribute to lower incidences of human diseases related to high dietary fat intake and stress (Weidmann, 2012).

**FIGURE 1:**
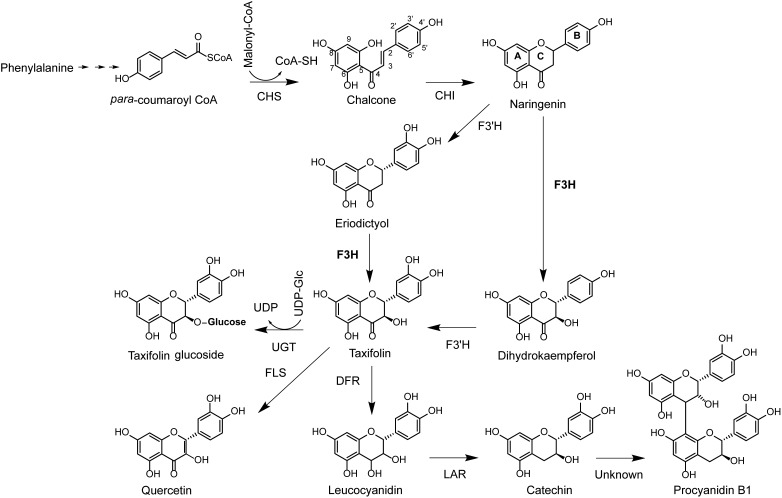
Biosynthesis pathway of the dihydrofavonol taxifolin and flavan-3-ols catechin and PAB1. CoA, coenzyme A; CHS, chalcone synthase; CHI, chalcone isomerase; F3H, flavanone 3-hydroxylase; F3′H, flavonol 3′-hydroxylase; FLS, flavonol synthase; DFR, dihydroflavonol reductase; LAR, leucoanthocyanidin reductase; UDP, uracil diphosphate; UGT, UDP-dependent glucosyl transferase.

Flavonoids are produced by sequential condensation of a phenylpropanoid acid coenzyme A (CoA) ester (C_6_–C_3_) to three malonyl CoA molecules to form chalcone with its typical C_6_–C_3_–C_6_ flavonoid backbone (Treutter, 2006). In the early steps of the flavonoid pathway, chalcone is modified via isomerization and oxidation to a dihydroflavonol (Winkel-Shirley, 2001). In most plants, dihydroflavonols are a major branch point in the flavonoid biosynthesis pathway, and subsequent reactions can either produce dihydroflavonol glucosides via glycosylation, flavonols or anthocyanidins via oxidation and flavan-3-ols via reduction (Figure 1).

The biosynthesis of dihydroflavonols was identified as a key regulatory point for the formation of down-stream metabolites in the flavonoid pathway and is catalyzed by flavanone 3β-hydroxylase (F3H; EC 1.4.11.9; Forkmann and Stotz, 1981). F3H has been isolated and characterized from more than 50 plant species (Meng et al., 2015; Han et al., 2017) and is a 2-oxoglutarate-dependent dioxygenase that catalyzes the 3β-hydroxylation of *2S*-flavanones to *2R,3R*-dihydroflavonols using 2-oxoglutarate, dioxygen (O_2_), ferrous iron, and ascorbate as substrates and co-factors (Britsch et al., 1992). Amino acid sequences of these enzymes are conserved, with well-defined 2-oxoglutarate and iron binding sites (Britsch and Grisebach, 1986; Britsch et al., 1992; Turnbull et al., 2004). The enzyme is localized in the cytoplasm (Tu et al., 2016) and may form multi-enzyme complexes with other enzymes involved in flavonoid biosynthesis (Burbulis and Winkel-Shirley, 1999). These multi-enzyme complexes allow for co-regulation of multiple enzymatic steps in the pathway. For example, *F3H* is transcriptionally regulated together with up-stream genes in *Arabidopsis thaliana* [*chalcone synthase* (*CHS*) and *chalcone isomerase* (*CHI*)] (Figure 1) (Pelletier and Shirley, 1996; Burbulis and Winkel-Shirley, 1999). However, in many other plant species, this gene is co-regulated with down-stream genes, including *dihydroflavonol reductase* (*DFR*), *anthocyanidin synthase* (*ANS*), and *leucoanthocyanidin reductase* (*LAR*) (Beritognolo et al., 2002; Singh et al., 2008; Liu et al., 2013; Song et al., 2016; Ullah et al., 2018).

*Flavanone-3-hydroxylase* gene expression is positively regulated by the plant hormones salicylic acid (Sun et al., 2016), jasmonic acid (Meng et al., 2015), and abscisic acid (Song et al., 2016). Increased transcription of the *F3H* gene leads to tolerance to abiotic as well as biotic stress, including drought (Watkinson et al., 2006), saline conditions (Mahajan and Yadav, 2014), cold (Meng et al., 2015), UV radiation (Liu et al., 2013) as well as biotrophic and necrotrophic attackers (Sun et al., 2016). High expression of this enzyme results in accumulation of proanthocyanidins (PAs), which are major end products of the pathway (Beritognolo et al., 2002; Song et al., 2016), higher levels of antioxidants (Meng et al., 2015) and lower levels of reactive oxygen species (Mahajan and Yadav, 2014).

Tree species within the Pinaceae, most notably spruce (*Picea* spp.) and pine (*Pinus* spp.) are economically important keystone species that dominate temperate, boreal, and montane landscapes. These long-lived woody perennials are very susceptible to the effects of climate change (Hanewinkel et al., 2013). Warmer weather conditions, wind storms and unseasonal frost have recently resulted in a world-wide decline of spruce and pine forests (Allen et al., 2010; Bentz et al., 2010). A main driver of these declines are bark beetles, which initially attack stressed and wind-damaged trees. This results in beetles building up massive population sizes and switch from an endemic population state to an epidemic phase. Bark beetles in the epidemic phase attack healthy trees by pheromone-driven mass attacks and disperse rapidly over wide areas resulting in the loss of millions of hectares of forest per year (Boone et al., 2011). Bark beetle success in overcoming the resistance mechanisms of healthy host trees has been partially ascribed to simultaneous attacks by bark beetle-associated fungi (Krokene, 2015), which are thought to exhaust tree defenses, although this view is not shared by others (Six and Wingfield, 2011).

In an effort to preserve Pinaceae forests in areas most affected by global warming, research is being conducted to identify resistance traits against bark beetles and their associated fungi (Keeling and Bohlmann, 2006; Hamberger et al., 2011; Krokene, 2015). These studies focused on understanding the biosynthesis of terpenoid oleoresin. Resins entrap and intoxicate attacking beetles and inhibit the growth of their fungal associates (Keeling and Bohlmann, 2006; Schiebe et al., 2012). The Pinaceae also produce high concentrations of polyphenols, such as stilbenes and PAs (Raiber et al., 1995; Booker et al., 1996; Li et al., 2012; Hammerbacher et al., 2011, 2013). Recent studies suggested that PAs (also known as condensed tannins) appear to function in tree defense against bark beetle-fungus invasions (Hammerbacher et al., 2014, 2018). However, little is known about the role of other flavonoids in the defense of spruce and pine against bark beetles and their associated fungi, although circumstantial evidence suggests that they should also play an important role (Brignolas et al., 1995; Li et al., 2012).

We therefore investigated the biosynthesis and defensive role of flavonoids in the Pinaceae using a study system of Norway spruce (*Picea abies*), its most important bark beetle pest (*Ips typographus*) and the bark beetle vectored fungus, *Endoconidiophora polonica*, an ascomycete that stains the wood with a blue color. We artificially simulated bark beetle attack by wounding trees and inoculating these wounds with *E. polonica*. Among the flavonoids, dihydroflavanol and flavan-3-ol content increased most significantly in the cambium of fungus-infected trees compared to wounded control trees. Bioassays of these compounds revealed that both classes were toxic to bark beetles as well as their fungal associates. Flavan-3-ol biosynthesis in Norway spruce was elucidated recently (Hammerbacher et al., 2014, 2018), but little is known about dihydroflavonol biosynthesis in this tree species. We identified a single gene encoding a functional F3H enzyme that produces dihydroflavonols in Norway spruce, which is transcriptionally up-regulated during simulated bark beetle attack. Suppressing expression of this gene by RNA interference (RNAi) in transgenic Norway spruce saplings resulted in lower concentrations of dihydroflavonols and flavan-3-ols, revealing its important role in regulating down-stream flavonoid accumulation in this species.

## Materials and Methods

### Simulated Bark Beetle Attack: Inoculation of Norway Spruce With Bark Beetle-Associated Fungus or Sterile Agar

Seven-year old Norway spruce saplings from the clone S21K0420232, purchased from Skogforsk (Sweden), were grown in 5 L pots in an outdoor plot at the MPI-CE, Jena, Germany as described previously (Hammerbacher et al., 2018). The saplings had a stem diameter of 2–2.5 cm. *E. polonica* isolate K2014 (*Kandasamy*, isol. Ex. bark beetle gallery, Thuringian Forest, Gotha, Germany), was grown on potato dextrose agar (Difco, BD, Franklin Lakes, NJ, United States) at 25°C for 7 days in darkness. In June 2015, a 5 mm diameter cork borer was used to remove a circular piece of bark from the lower stems (approximately 80 cm above ground level) of the spruce saplings. A similar-sized disk of the fungal culture was inserted into the wound and sealed with Parafilm. Sterile disks of potato dextrose agar were used for control treatments. In total 20 saplings were inoculated and 20 were wounded. Actively growing cambium from five trees (*n* = 5) from inoculated and wounded saplings were harvested after 2, 7, 14, and 28 days post inoculation (dpi) and flash frozen in liquid nitrogen. Sections from 2.5 cm above to 2.5 cm below the inoculation point were harvested from all treatments at 2 and 7 dpi as well as from the sterile agar-inoculated treatments at 14 and 28 dpi. The fungus-inoculated lesions from the 14 and 28 dpi treatments were separated into two samples, comprising (1) a section from 2.5 cm above to 2.5 cm below the point of inoculation (inner lesion) and (2) sections from 2.5 to 4 cm both above and below the point of inoculation (outer lesion).

### Flavonoid Analysis

Samples from fungus-inoculated treatments and sterile agar-inoculated controls as well as stems of transgenic spruce carrying the F3H RNAi construct were finely ground in liquid nitrogen using a mortar and pestle. A subsample of the resulting wood powder was lyophilized at 0.34 mbar pressure using an Alpha 1-4 LD plus freeze dryer (Martin Christ GmbH, Osterode, Germany). Approximately 20 mg of dried spruce tissue powder was extracted twice for 4 h with 800 μl analytical grade methanol containing 10 μg ml^-1^ internal standard, apigenin-7-glucoside (Carl Roth GmbH, Karlsruhe, Germany). Flavonoids were analyzed by LC-tandem mass spectrometry on an Agilent 1200 HPLC system (Agilent, Santa Clara, CA, United States) coupled to an API 3200 mass analyzer (Sciex, Darmstadt, Germany) using the protocols described in Hammerbacher et al. (2014) for the following analytes: quercetin-glucoside, catechin, gallocatechin, procyanidin B1, and catechin-gallocatechin dimers. Dihydromyricetin was measured using the protocols from Hammerbacher et al. (2018). Additional multiple reaction monitoring (MRM) was used in this study to measure analyte precursor ion → fragment ions for additional flavonoid metabolites as follows: *m/z* 271.0 → 151.0 [collision energy (CE), -28 V; declustering potential (DP), -55 V] for naringenin; *m/z* 287.0 → 151.0 (CE, -20 V; DP, -75 V) for eriodictyol*; m/z* 303.0 → 125.0 (CE, -28 V; DP, -40 V) for taxifolin; *m/z* 465.0 → 285.0 (CE, -30 V; DP, -70 V) for taxifolin glucoside. To develop the MRMs, standards were purchased from Sigma-Aldrich (naringenin, eriodictyol, and taxifolin), or from TransMIT GmbH Giessen, Germany (taxifolin-glucoside, dihydromyricetin). Products of *in vivo* conversion of naringenin and eriodictyol by *PaF3H* in *Escherichia coli* were analyzed using a Bruker Daltronics ion trap mass spectrometer following the protocols of Hammerbacher et al. (2014). External calibration curves from 1 μg ml^-1^ to 1 mg ml^-1^ were used for quantification of all metabolites reported in this study, except for taxifolin glucoside for which a calibration curve of quercetin rhamnoside (TransMIT) was used with a response factor of 1.

### Identification of *Pa*F3H Candidate Sequences and Phylogenetic Analysis

Protein sequences of previously characterized F3H genes were downloaded from NCBI and used in BLASTP searches against all *Picea* transcript assemblies in the conifer genome database^1^ and on NCBI. Transcripts with high identity to known F3H sequences from *P. abies*, *P. glauca*, and *P. sitchensis* were assembled into full-length contigs using CLC Workbench (Qiagen, Aarhus, Denmark). Primers were designed for the 5′ and 3′ ends of the full length transcript of *P. abies* (F3HF GGGGACAAGTTTGTACAAAAAAGCAGGCTCAATGGCGCCCGCCGCAGTCGTGG and F3HR GGGGACCACTTTGTACAAGAAAGCTGGGTATCACGACTTTTCCTGCTCAGCAAC). RNA was extracted from approximately 50 mg of fresh, frozen (-80°C) spruce tissue as described in Hammerbacher et al. (2018) using the Stratec Plant RNA mini kit (Stratec, Birkenfeld, Germany). RNA quality was assessed spectrophotometrically and by agarose gel electrophoresis. RNA samples with a concentration higher than 100 ng μl^-1^ and an A_260/280_ higher than 1.75 was used for reverse transcription. Approximately 500 ng total RNA was reverse-transcribed to cDNA using Superscript v. II (Invitrogen) following the manufacturer’s protocols. Full length *PaF3H* was then amplified from cDNA using Phusion Taq (New England Biolabs, Ipswich, MA, United States) and cloned into the Gateway compatible vector pDONR 207 (Invitrogen, Carlsbad, CA, United States) using BP clonase II (Invitrogen). To verify the *PaF3H* sequence, the entry clones were sequenced using pDONR F and R primers (Invitrogen) using Sanger chain termination sequencing and the BigDye v3.1 cycle sequencing kit (Thermo Fisher Scientific) and an ABI prism 7000 capillary electrophoresis sequencing instrument (Thermo Fisher Scientific). Protein sequences of characterized F3H enzymes from different angiosperm species were aligned with the translated nucleotide sequences of *F3H* from different gymnosperm species (Supplementary Figure S1) using the MAFFT web interface^2^ and imported into MEGA v.6 (Kumar et al., 2008; Center for Evolutionary Medicine and Informatics, Tempe, AZ, United States). The evolutionary history was inferred by using the Maximum Likelihood method based on the JTT matrix-based model (Jones et al., 1992). The tree with the highest log likelihood (-10349.13) was constructed (500 boot strap replicates). The percentage of trees in which the associated taxa clustered together is shown next to the branches. Bootstrap values lower than 50% are not shown. The initial tree for the heuristic search was obtained automatically by applying Neighbor-Join and BioNJ algorithms to a matrix of pairwise distances estimated using a JTT model, and then selecting the topology with a superior log likelihood value. The tree is drawn to scale, with branch lengths measured as the number of substitutions per site. The analysis involved 22 amino acid sequences. All positions containing gaps and missing data were eliminated. There were a total of 262 positions in the final dataset (GenBank accession numbers for all sequences are included in Supplementary Table S1).

### qPCR

RNAs were extracted and reverse transcribed as described above from the same samples from which the metabolites were extracted. Approximately 100 ng cDNA were used per PCR reaction. qPCR was performed with the Kappa SYBR Fast qPCR kit (Kappa Biosystems, Boston, MA, United States) and a CFX96 – Real-Time System (Bio-Rad, Hercules, CA, United States) following the manufacturer’s specifications. *PaF3H* was amplified using PaF3H-F (GCAGAGCGTGCACAG) and PaF3H-R (GTGAGTTGAGTTCTGTGGAG) primers with an initial denaturation step at 95°C for 2 min. followed by 40 cycles of 95°C denaturation and 60°C extension. The PCR was normalized using *PaUBI* (Schmidt et al., 2010; GB:EF681766.1) and calibrated to the 2 days sterile agar-inoculation treatment. The means of three technical replicates from each of five biological replicates per treatment group were used to calculate manually the relative transcript abundance according to Pfaffl (2001). A dilution series was made to determine the primer efficiencies, which were close to 100%. Non-template controls and non-reverse transcribed RNA controls were included. Each PCR product was analyzed using a melting curve to verify single product amplification.

### Functional Characterization of *Pa*F3H by Expression in *E. coli* and RNAi in Transgenic Norway Spruce

The *in vivo* enzyme activity of PaF3H was assessed by transforming *E. coli* BL21 with *PaF3H* cloned into the destination vector pDEST15 (Invitrogen) using LR clonase (Invitrogen) following the manufacturer’s protocols. BL21 (DE3) bacterial cells were grown on Luria-Bertani (LB) plates containing 100 μg ml^-1^ ampicillin. For protein expression single colonies were inoculated into 5 ml LB broth with 100 μg ml^-1^ ampicillin, and grown for 12 h at 30°C. The 5 ml starter cultures were used to inoculate 100 ml LB medium supplemented with 100 μg ml^-1^ ampicillin. Bacterial cultures were grown for 9 h at 18°C (220 rpm) and protein expression was induced with 0.5 mM IPTG (isopropyl β-D-1-thiogalactopyranoside). Two hours after inducing expression with IPTG the cultures were split and naringenin or eriodictyol in DMSO was added to the culture medium to a final concentration of 200 μg ml^-1^. Cultures were harvested 12 h after addition of the substrates. Bacteria were removed from the culture medium by centrifugation. The medium was acidified with 1% (v v^-1^) of 0.1 N HCl and extracted with three volumes of ethyl acetate. The ethyl acetate extracts were evaporated using a rotary evaporator and re-dissolved in 500 μl methanol for LC-MS analysis following the protocols described above.

In order to assess the *in planta* activity of *Pa*F3H, transgenic saplings were made carrying an *F3H* RNAi construct. For that a 330-bp region of the *PaF3H* gene was PCR-amplified by using the oligonucleotides *PaF3HRNA*i-for (tgctctagagcagggtttggcatgcaatatgttggcg) and *PaF3HRNA*i-rev (cgggatcccgggtccgcgctcttgaactt) and cloned in sense and antisense orientations into the multiple cloning sites of the pTRAIN vector on either side of an intron as described by Levée et al. (2009). After HindIII digestion, the excised RNAi-cassette including also an upstream maize ubiquitin promotor was ligated into the multiple cloning site of the pCAMBIA 1305.2 vector^3^. *Agrobacterium tumefaciens*-mediated stable transformation of *P. abies* embryogenic tissue (Pa186/3c) was performed as described in detail by Schmidt et al. (2010). Generation of somatic transgenic seedlings was based on a protocol originally reported for white spruce from Klimaszewska et al. (2005). Seedlings of three independent kanamycin-resistant transgenic lines plus an empty vector control line were characterized by qPCR, using four plants per line. Transgenic seedlings were grown for 2 years before the stem tissue was harvested, ground to a fine powder under liquid nitrogen and analyzed using both qPCR and LC-MS (Supplementary Figure S2).

### Fungal Growth on Catechin and Taxifolin

Growth of *E. polonica* on potato dextrose medium containing either DMSO without flavonoids (control), 2 mg ml^-1^ catechin, 2 mg ml^-1^ taxifolin, or 2 mg ml^-1^ of a physiologically relevant 2:1 mixture of catechin and taxifolin was determined in Petri dishes using the protocols described by Hammerbacher et al. (2014). In brief, the compounds were added to the medium in DMSO (50 mg ml^-1^) just before the medium was dispersed into Petri dishes. Fungal growth of four replicates per compound was measured daily until the fungus grew to the edges of the Petri dish. Fungal growth was plotted for each Petri dish and a linear curve was fitted. The growth rate was determined by the gradient of the fitted curve.

### Bark Beetle Feeding Assays

Adult bark beetle feeding assays were performed in 12 cm long pneumatic tubes, outer diameter 6 mm, inner diameter 4 mm (Sang-A Pneumatic, Daegu, Korea). Each assay tube was sealed at one end with Parafilm and filled with spruce bark diet prepared as follows: 7% (w/v) finely milled spruce inner bark powder was mixed with 1% fibrous cellulose (Sigma), 4% Bactoagar (Roth) in water and autoclaved for 20 min at 121°C. In order to facilitate uniform packing of the diet in each tube, the diet mix was vortexed thoroughly and drawn up in a disposable sterile syringe. The end of the syringe was fitted onto the open end of the assay tubes to discharge the diet into them. The open ends of the assay tubes were then sealed and the medium was allowed to dry overnight at room temperature. Approximately 2 cm of diet was manually removed from one end of the assay tube to facilitate insertion of bark beetles. Stock solutions of catechin and taxifolin were prepared in DMSO and added to the spruce bark agar before filling the mixtures into the tubes. Only DMSO was added in the control diet without amended compounds. The final concentrations of the compounds in the medium were 1 mg ml^-1^ catechin, 1 mg ml^-1^ taxifolin, or 0.5 mg ml^-1^ of both catechin and taxifolin (1:1). Adult beetles used in this assay were reared in the laboratory using the protocols of Anderbrant et al. (1985). The initial insects for the laboratory colony were collected from naturally infested trees in Jena, Germany. The weight of each beetle was measured to the nearest 0.01 mg, and it was placed carefully in the assay tube with its head facing towards the diet. The length of the feeding tunnel in each tube was measured after 6 and 48 h and the weight of the beetle was then measured after 48 h to determine the weight change resulting from different treatments. Previous studies have reported 6 h to be sufficient to see the difference in tunneling length and 48 h for weight change in adult bark beetles (Faccoli and Schlyter, 2007, Cardoza et al., 2008). Each treatment (control, catechin, taxifolin, or the mixture of both compounds) was replicated 15 times. The assay beetles included both males and females.

### Statistical Analysis

All data were subjected to a Shapiro–Wilk test for normality and non-normal data were transformed with the log or (1 + log) function. Transformed or non-transformed data were analyzed using two- or one-way ANOVAs or repeated measures ANOVA (specifically for the tree inoculation trial). Following this, Tukey’s *post hoc* test was used on data showing significant differences using a 95% confidence interval. For bark beetle feeding assays, a paired sample *t*-test was used to compare the mean weight of beetles before and after feeding. All analyses were conducted using R^4^.

## Results

### Flavonoids Accumulate in Norway Spruce Upon Infection With the Bark Beetle–Associated Fungus, *E. polonica*

Norway spruce saplings were wounded and inoculated with the bark beetle-associated, sapwood-staining fungus *E. polonica* or inoculated with sterile agar as a control. Samples were harvested from both infected and control trees for analysis over a 28 days time course. Fungal lesions at 14 and 28 days post-inoculation (dpi) were separated into two sections: The inner lesion section contained fungus that established itself at the onset of the experiment and the outer lesion section was made up of newly infected tissue. Control samples equivalent to the inner lesion region were harvested at the same time points. Flavanones (naringenin and eriodictyol), dihydroflavonols (taxifolin, taxifolin-glucoside, and dihydromyricetin), flavonols (quercetin, myricetin, laricitrin, and their glycosides) as well as flavan-3-ols (catechin, gallocatechin, and dimeric proanthcyanidins) were analyzed in the cambial tissues using high-performance liquid chromatography coupled to mass spectrometry (LC-MS). Among these naringenin, eriodictyol, taxifolin, taxifolin glucoside, catechin, and procyanidin B1 could be detected above the quantification limit (Supplementary Figure S3 and Supplementary Table S2).

**FIGURE 2:**
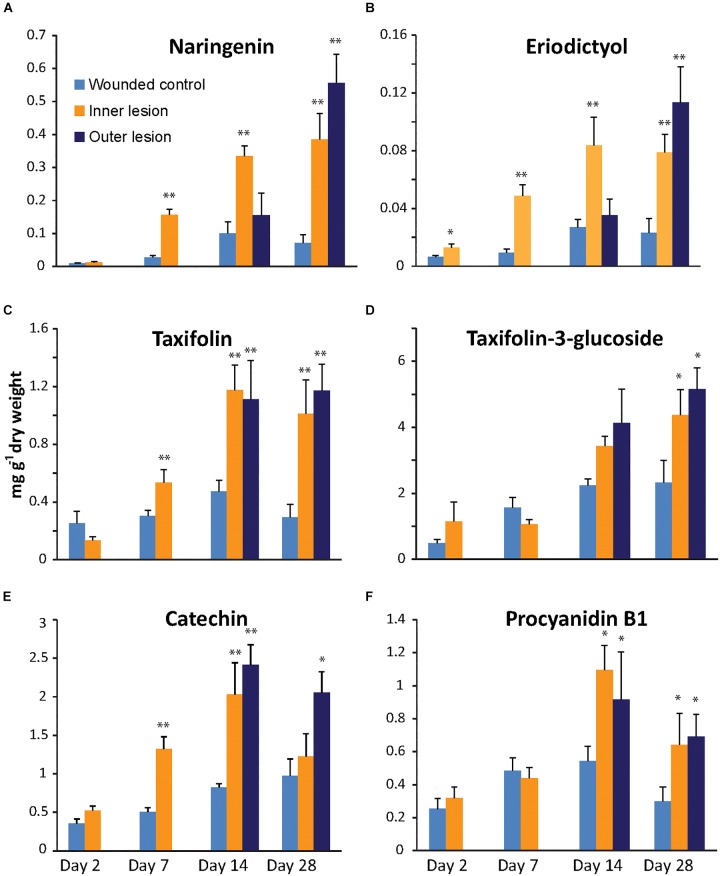
Accumulation of flavanones **(A**,**B)**, dihydroflavonols **(C**,**D)** and flavan-3-ols **(E**,**F)** in the cambial region of Norway spruce saplings during infection by *Endoconidiophora polonica* compared to control saplings inoculated with sterile agar. Naringenin **(A)**, eriodictyol **(B)**, taxifolin **(C)**, taxifolin-3-glucoside **(D)**, catechin **(E)** and procyanidin B1 **(F)** contents were measured by LC-tandem mass spectrometry. Statistical significance against the wounded control treatment is shown (significance codes: ^∗^*p* < 0.04; ^∗∗^*p* < 0.01; *n* = 5; error bars = SE).

The flavanone naringenin, as well as its 3′-hydroxylated derivative eriodictyol (Figure 1) accumulated to significantly higher concentrations in the inner lesions compared to the wounded control (*p* < 0.001) at 7 dpi and reached a maximum at 14 dpi after which the content of both flavanones remained constant (Figure 2A,B). In the newly infected tissue of the outer lesion, the concentration of naringenin and eriodictyol only increased significantly above control concentrations at 28 dpi (*p* < 0.001). A slight increase in the concentrations of both flavanones was also observed in the controls inoculated with sterile agar over the time course of 28 days, which was significant between 7 and 14 dpi (*p* < 0.01).

The dihydroflavonol taxifolin (*2R,3R*-dihydroquercetin) and its glycosylated derivative, taxifolin-3-glucoside were detected at much higher concentrations than the flavanones. The glucoside accumulated to levels as high as 5 mg g^-1^ dry weight after 28 dpi in fungus-infected tissue (Figure 2C,D). The aglucone accumulated more rapidly in fungus-inoculated tissue than the glucoside and reached significantly higher levels than in the controls as early as 7 dpi (*p* < 0.01). Taxifolin reached a maximum in both inner as well as outer lesions at 14 dpi and remained constant until 28 dpi. Compared to the aglucone, the glucoside accumulated at a slower rate, and reached a maximum at 28 dpi, which was significantly higher than the levels in the control (*p* < 0.05) in both the inner as well as in the expanding outer lesion. An increase of taxifolin-3-glucoside was also recorded in the controls inoculated with sterile agar over the time course of this experiment, where treatments at 2 dpi differed significantly from treatments at 28 dpi (*p* < 0.05).

The last two flavonoids detected in our analysis were both flavan-3-ols that are precursors for the biosynthesis of condensed tannins (Figure 2E,F). Monomeric catechin could be detected at higher levels than its dimerized derivative procyanidin B1 (PAB1) in both uninfected and infected spruce stems. In the inner lesion, catechin content increased significantly above the control by 7 dpi (*p* < 0.001) reaching a maximum at 14 dpi, similar to the flavanones and taxifolin, but leveled off close to control levels at 28 dpi. In the expanding outer lesion, on the other hand, significantly higher levels of catechin were detected at 14 and 28 dpi compared to the control. Catechin concentrations in the sterile agar control increased during the 28 days of this experiment and were significantly higher at 14 dpi compared to 2 dpi (*p* < 0.01). Dimeric PAB1 content in *E. polonica* inoculated tissue did not increase as dramatically as catechin content, but also reached significantly higher levels compared to the control (*p* < 0.05). However no significant increases were observed in the control over time.

Taken together, these data show that the flavonoid content of spruce stems increases in response to fungal infection. Monomeric aglucone concentrations increased more rapidly and to higher levels in fungus-infected tissue relative to the sterile agar-inoculated controls. Monomers and aglucones also increased more rapidly compared to their glycosylated and dimeric derivatives. Among the aglucones, the content of the dihydroflavonol taxifolin and the flavan-3-ol catechin were significantly higher compared to that of the flavanones, naringenin and eriodictyol.

### A Physiologically Relevant Mixture of the Flavonoids Taxifolin and Catechin Is More Toxic to *E. polonica* Than the Single Compounds

**FIGURE 3:**
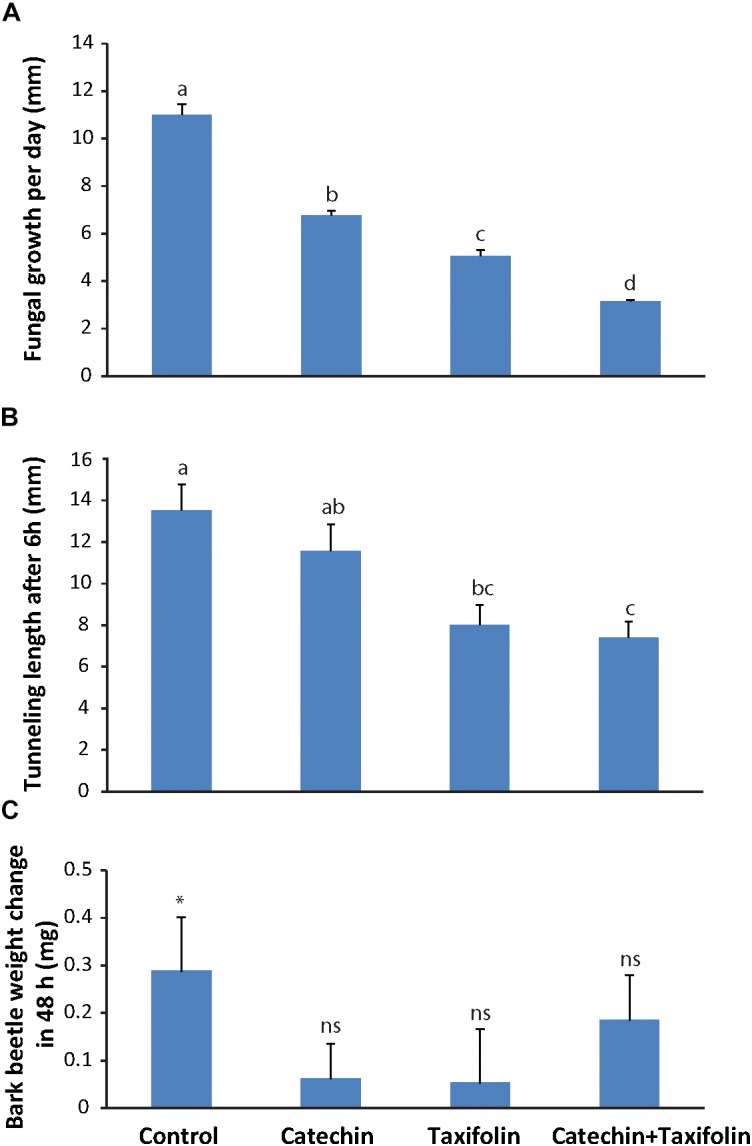
Taxifolin and catechin are defense compounds in Norway spruce that act against both bark beetles and an associated fungus. **(A)** *E. polonica* grew significantly slower on potato dextrose agar amended with 2 mg ml^-1^ catechin, 2 mg ml^-1^ taxifolin, or a 2 mg ml^-1^ mixture of catechin and taxifolin (2:1) (*n* = 4; error bars = SE; *p* < 0.001). **(B)** *Ips typographus* tunneled significantly further into bark medium containing 1 mg m^-1^ catechin or unamended medium compared to medium with a 1:1 catechin-taxifolin mixture (*n* = 15; error bars = SE; *p* < 0.001). **(C)** Bark beetles gained more weight feeding on unamended bark medium without flavonoids than on medium amended with 1 mg ml^-1^ catechin, taxifolin, or both (*n* = 15; error bars = SE; *p* < 0.05). Different letters refer to statistically significant differences at *p* < 0.05.

Due to their high and rapid accumulation in fungus-infected spruce tissue, the anti-fungal activity of catechin, and taxifolin was assessed in an *in vitro* assay. Potato dextrose agar, allowing for optimal growth of *E. polonica* (negative control), was amended with either 2 mg ml^-1^ catechin, 2 mg ml^-1^ taxifolin, or 2 mg ml^-1^ of a 2:1 mixture of catechin and taxifolin (similar to ratios observed in the fungus-inoculated treatments, Figure 2). Fungal growth was measured daily and the ultimate growth rate was calculated from the gradient of the growth curve (Figure 3A).

Both catechin and taxifolin significantly inhibited fungal growth. However, taxifolin was more toxic to the fungus than catechin. Interestingly, a mixture of both compounds in amounts equivalent to what was observed in fungus-induced spruce tissue (Figure 2) was even more toxic to *E. polonica* than the individual compounds alone (*p* < 0.001; Figure 3A). Catechin and taxifolin therefore have a synergistic antifungal effect. Based on these data we hypothesize that taxifolin plays an important role in the chemical defense of Norway spruce against infection by the bark beetle associated fungus *E. polonica*.

### Catechin and Taxifolin Are Toxic to Bark Beetles and Inhibit Tunneling

To assess if catechin and taxifolin have an effect on *I. typographus*, an assay was conducted where bark beetles were forced to tunnel into medium containing either 1 mg ml^-1^ catechin, taxifolin, or 0.5 mg ml^-1^ of both compounds (similar to ratios observed in the wounded control treatments, Figure 2). After 6 h, the tunnel lengths of beetles feeding either on taxifolin or a 1:1 mixture of taxifolin and catechin were significantly shorter (*p* < 0.001) than those of beetles feeding on unamended control medium or on catechin (Figure 3B). This result suggests that taxifolin is an anti-feedant for *I. typographus*. Beetles were allowed to continue tunneling and feeding for 48 h before they were weighed. Beetles that fed on non-amended control diet gained significant weight after feeding for 48 h (pairwise *t*-test before and after feeding for 48 h on diet: *t*_14_ = -2.43, *p* = 0.03). Beetles feeding on either catechin or taxifolin amended diets did not gain weight (pairwise *t*-tests: catechin: *t*_13_ = -0.84, *p* = 0.42, taxifolin: *t*_14_ = -0.47, *p* = 0.65). Interestingly, insects feeding on the diet amended with the mixture of catechin and taxifolin gained weight, but the weight gain was not statistically significant (pairwise *t*-test: *t*_14_ = -1.89, *p* = 0.08; Figure 3C). These results suggested that the added compounds had an anti-nutritive effect and that weight gain was affected mainly by the concentration of individual compounds rather than the composition of mixtures.

### Taxifolin Is Synthesized by a Flavanone-3-Hydroxylase Enzyme, Which Is Encoded by a Single Gene in Norway Spruce and Is Highly Expressed in Response to Fungal Infection

The biosynthesis of catechin is well studied in Norway spruce (Hammerbacher et al., 2014), but little is known about the formation of taxifolin in this species. It is known that dihydroflavonols, such as taxifolin, are synthesized from flavanones by 2-oxoglutarate dependent dioxygenases known as flavanone 3-hydroxylase (F3H) enzymes. Only one putative protein sequence with high identity to other characterized F3H enzymes could be retrieved from the *P. abies* High Confidence Genes database^5^. On the other hand, Warren et al. (2015) annotated five open reading frames in the *P. glauca* genome as F3H-encoding genes. We manually re-annotated these genes and eliminated four of them, which encoded enzymes with high similarity to other oxoglutarate-dependent dioxygenase enzymes, including a flavonol synthase as well as an anthocyanidin reductase enzyme. Furthermore, none of the inferred protein sequences from Warren et al. (2015) corresponded to the transcript sequences deposited on NCBI for *P. sitchensis* (Ralph et al., 2008), or *P. abies*^5^, both close relatives of *P. glauca*. BLAST searches of available transcriptomes of *P. abies, P. glauca, P. sitchensis, Pinus radiata*, and *P. taeda* using characterized F3H protein sequences from tea (Singh et al., 2008), apple (Halbwirth et al., 2002), and petunia (Britsch et al., 1992) F3H protein sequences resulted in the retrieval of a single high-confidence F3H transcript for each species.

**FIGURE 4:**
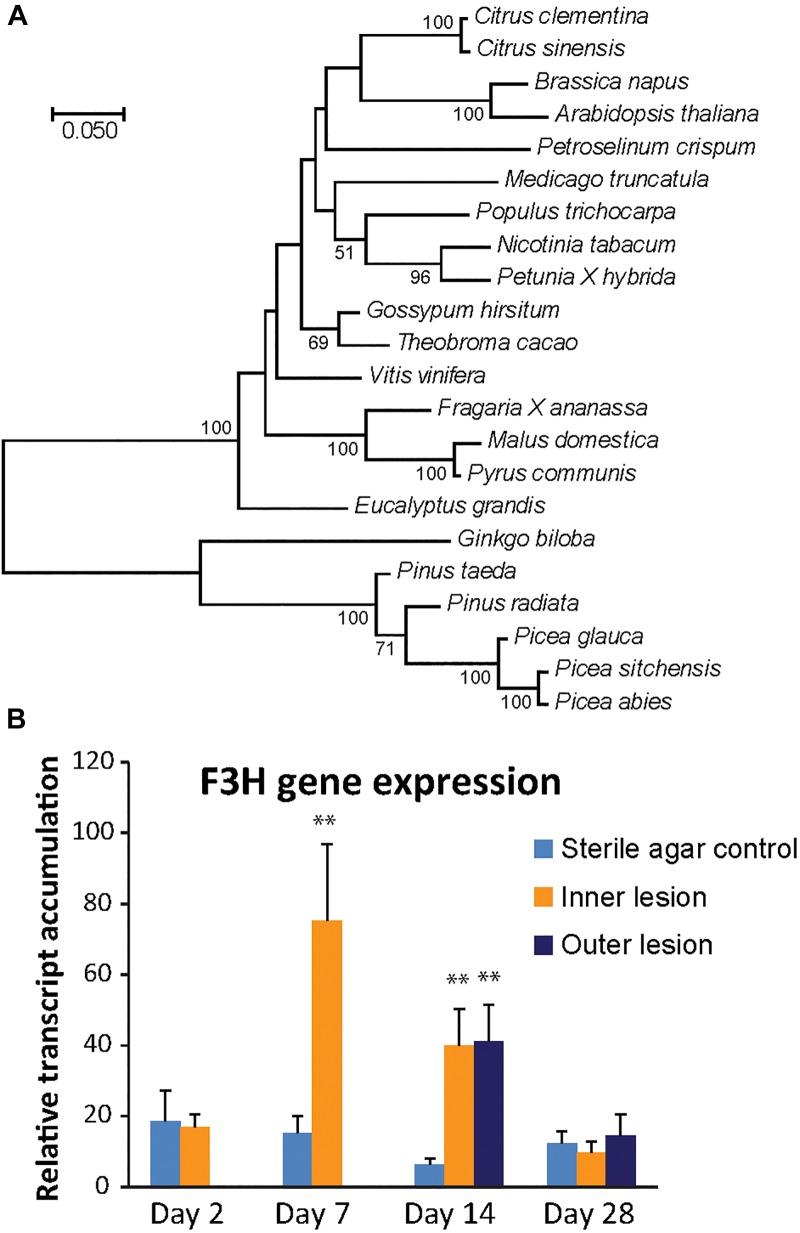
The *Pa*F3H gene encodes the enzyme for the production of taxifolin in Norway spruce. **(A)** Phylogenetic analysis revealed separate clades for F3H enzymes from gymnosperms and angiosperms (500 boot-strap replicates). Scale bar shows number of substitutions per site. **(B)** F3H gene expression increased significantly 7 and 14 days post inoculation compared to the wounded control (significance code: ^∗∗^*p* < 0.01; *n* = 5; error bars = SE). Transcript abundance was measured by qRT-PCR normalized against *PaUBI* and calibrated against abundance in a sterile agar-inoculated control sample. Different letters refer to statistically significant differences at *p* < 0.05.

Phylogenetic analysis of the putative *P. abies* F3H protein and other F3H proteins from both angiosperms and gymnosperms revealed a clear separation of F3H protein sequences from the Pinaceae. F3H from the Pinaceae formed a clade with *Gingko biloba*, which is also a gymnosperm. *P. abies* F3H was 99% similar to *P. sitchensis* and 96% similar to the *P. glauca* F3H enzyme, but only 89% similar to the F3H enzymes from the two *Pinus* species included in the analysis and 84% similar to that from *G. biloba*. Angiosperm F3H sequences were clearly separated from the gymnosperm sequences and had on average 66–70% amino acid identity to the F3H sequence from *P. abies* (Figure 4A).

The expression of the candidate *PaF3H* gene was analyzed in fungus-inoculated and sterile agar inoculated-control tissue using quantitative real-time PCR (Figure 4B). Compared to the saplings inoculated with sterile agar, a significant increase in transcript abundance was observed in fungus-inoculated tissue 7 and 14 dpi in the inner lesion (*p* < 0.01). The outer lesion showed an increase in transcription at 14 dpi, which was equivalent to that observed for the inner lesion. The *PaF3H* gene expression correlated well with taxifolin accumulation in the same tissues (Figure 2C), which increased at 7 dpi in the inner lesion above control levels and reached a maximum at 14 dpi in both inner and outer lesions. This supported our hypothesis that the *PaF3H* gene is involved in the biosynthesis of taxifolin in defense-induced Norway spruce tissue.

### *PaF3H* Encodes a Functional Flavanone 3-Hydroxylase Enzyme and Is Involved in Taxifolin Biosynthesis in Norway Spruce

**FIGURE 5:**
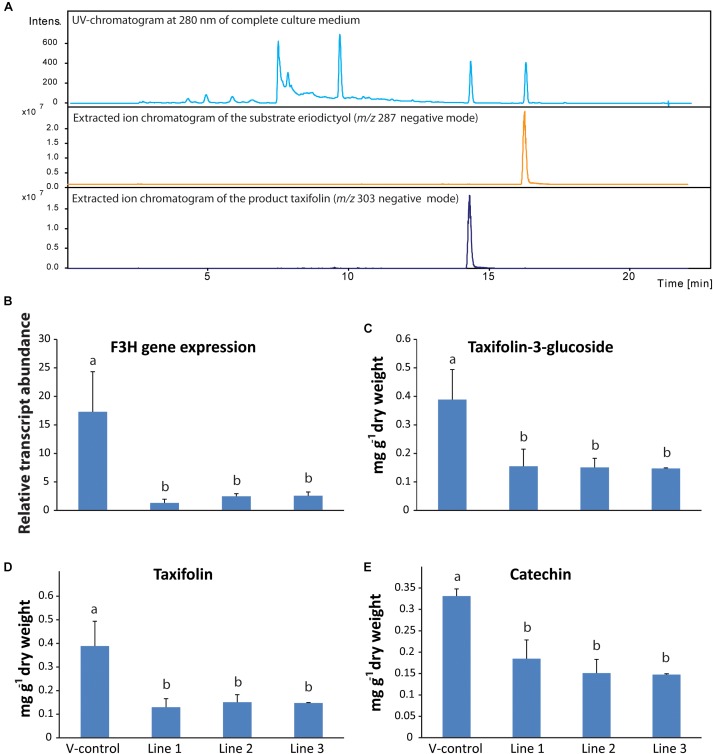
Functional characterization of flavanone-3-hydroxylase gene in Norway spruce. **(A)** *Pa*F3H expressed heterologously in *Escherichia coli* produced taxifolin when the substrate eriodictyol was fed to the culture. RNA-interference of F3H led to reduced levels of *PaF3H* transcripts **(B)**, as well as reduced levels of taxifolin **(C)**, taxifolin glucoside **(D)** and catechin **(E)** in transgenic trees compared to the empty vector control (*n* = 4; error bars = SE; *p* < 0.05). Transcript abundance was measured by qRT-PCR normalized against *PaUBI* and calibrated against the abundance of RNAi line 1. Metabolites were analyzed by LC-tandem mass spectrometry.

The open reading frame of *PaF3H* was cloned and the encoded protein was heterologously expressed in *E. coli*. The substrates naringenin or eriodictyol were added to the medium to a final concentration of 0.2 mg ml^-1^ 2 h after induction of protein expression using IPTG. 12 h after adding the substrate to the growth medium, it was extracted with acidic ethyl acetate and analyzed using LC-MS. Addition of naringenin to the medium resulted in the accumulation of dihydrokaempferol (data not shown) and addition of eriodictyol resulted in taxifolin accumulation (Figure 5A). Medium-only as well as non-transformed *E. coli* controls were included in the assays. Peak identities and retention times were confirmed with pure standards. *PaF3H* therefore encodes an enzyme, which can accept both naringenin and eriodictyol as substrates (Supplementary Table S3).

To study the *in planta* role of *PaF3H*, an RNA-interference (RNAi) construct was made for this gene and Norway spruce callus was transformed with it. Transgenic calli were selected and induced to differentiate into saplings, which were analyzed for their flavonoid content. Three transgenic lines were obtained with active expression of the RNAi construct. Due to the RNAi constructs, transcript abundance of the *PaF3H* gene was around 4- to 8-fold lower in the transgenic lines compared to the trees transformed with the vector only (V-control; Figure 5B). Consequently the accumulation of taxifolin (Figure 5C), taxifolin-3-glucoside (Figure 5D) and catechin (Figure 5E) was significantly lower in the transgenic F3H-RNAi lines than in the vector control line (*p* < 0.01). Taken together, these data show that *PaF3H* encodes a functional F3H, which contributes significantly to the accumulation of taxifolin as well as down-stream products of the pathway, including taxifolin-3-glucoside and catechin.

## Discussion

Due to climate change, conifers adapted to cool, wet conditions, such as Norway spruce, are currently experiencing increasing heat and drought stress (Netherer et al., 2015). This renders them susceptible to attack by bark beetles and their fungal associates, which can cause extensive forest losses under warm and dry conditions (Krokene, 2015). To offset climate-driven tree mortality, efforts are underway to identify resistance mechanisms in conifers that may render them partially tolerant to insect and pathogen attacks (Keeling and Bohlmann, 2006; Schiebe et al., 2012). In our study, we focused on Norway spruce, which is a host for the bark beetle *I. typographus* and its fungal associates. This tree species produces terpenoid oleoresins and polyphenols in response to bark beetle-fungus attacks (Schiebe et al., 2012), but which compounds are important in warding off such attacks is still unclear. To address this question systematically, we focused in this study on flavonoid biosynthesis in Norway spruce and the bioactivities of the most abundant flavonoids produced by the tree during simulated bark beetle attack.

### Two Classes of Flavonoids, Dihydroflavonols and Flavan-3-ols, Accumulate in the Cambial Zone of Norway Spruce After Fungal Infection

In addition to acetophenones, hydroxycinamic acids, coumarins, lignans, neolignans, and stilbenes, Norway spruce produces a diverse range of flavonoids, especially in foliage. Strack et al. (1989) showed, using nuclear magnetic resonance spectroscopy, that spruce needles contained nine different flavonoids, including the flavan-3-ols catechin and gallocatechin, the glucosides of kaempferol, quercetin, and isorhamnetin as well as the dihydroflavonol taxifolin. In addition, Slimestad and Hostettmann (1996) analyzed spruce needles with LC-MS and found the glucosides of myricetin, syringetin, laricitrin and taxifolin, rutinosides of kaempferol, quercetin, myricetin and syringetin as well as acylated glucosides of kaempferol, quercetin, laricitrin, syringetin and isorhamnetin. However, the diversity of flavonoids in bark and wood seems more limited. Only the flavonols quercetin, kaempferol, laricitrin, myricetin and their glucosides, the dihydroflavonol taxifolin and its glucoside as well as a diverse range of flavan-3-ols have thus far been identified in the bark of spruce trees in significant concentrations (Brignolas et al., 1995; Li et al., 2012; Schiebe et al., 2012; Hammerbacher et al., 2014, 2018). In this study we specifically analyzed the flavonoids in developing xylem and phloem and detected high levels of taxifolin, its glucoside, catechin and its dimeric derivative PAB1 as well as their metabolic precursors, naringenin and eriodictyol. Interestingly, concentrations of all these compounds increased significantly after simulated bark beetle attack compared to the wounded control.

The flavanones naringenin and eriodictyol are metabolic precursors for down-stream metabolites in the flavonoid pathway (Figure 1) and thus only accumulated at concentrations that were three to fifteen times lower than the other flavonoids identified in our study. These flavanones could possibly play an important role in Norway spruce defense against bark beetles and their associated fungi, for example as precursors. However, due to their low concentrations and due to the fact that bark beetles were reported to carry bacterial symbionts that can degrade them (Cheng et al., 2018) we did not study their direct defensive properties against *I. typographus* and its associated fungus *E. polonica*.

Two of the major end products of the flavonoid pathway in Norway spruce are flavan-3-ols, the monomer catechin and the catechin-epicatechin dimer, PAB1 (Hammerbacher et al., 2014). We found that these compounds accumulated in response to inoculation with the bark beetle associated fungus *E. polonica*, which is in agreement with previous studies (Brignolas et al., 1995; Hammerbacher et al., 2014). Interestingly, a strong and rapid accumulation of these compounds in Norway spruce correlated with resistance to both bark beetle attack (Brignolas et al., 1995) as well as spruce rust (*Chrysomyxa rhododendri*) infection (Ganthaler et al., 2014). Furthermore, an allele of *leucoanthocyanidin reductase 3* (*PaLAR3*), one of the four genes encoding the enzyme catalyzing the conversion of leucoanthocyanidin into catechin, was identified as a quantitative trait locus in Norway spruce accounting for 27% lower growth of *Heterobasidion* root rot fungus in sapwood (Nemesio-Gorriz et al., 2016). All this evidence therefore suggests that elevated catechin biosynthesis is a general anti-fungal defense in Norway spruce.

Another class of flavonoids, dihydroflavonols, is also induced by fungal attack on Norway spruce. We showed that high concentrations of taxifolin and its glucoside accumulated in response to infection with a bark beetle-associated fungus, as would happen during a bark beetle attack. Norway spruce was also shown to accumulate taxifolin in response to low density *E. polonica* infection (Evensen et al., 2000; Krajnc et al., 2014) as well as during infection by the spruce rust, *C. rhododendri* (Ganthaler et al., 2014). Similarly, other conifer species were also shown to accumulate taxifolin in response to fungal infection. For example, in Sitka spruce, increased taxifolin content was recorded after *Heterobasidion annosum* infection (Deflorio et al., 2011), and *Pinus nigra* responded to inoculation with *Diplodia sapinea* by producing both taxifolin and catechin (Bonello and Blodgett, 2003). The constitutive levels of taxifolin may also be important in spruce-fungus interactions since high constitutive levels of taxifolin glucoside content correlated positively with resistance to spruce rust (Ganthaler et al., 2014), and the absence of this compound was thought to partially explain spruce susceptibility to bark beetle attack (Brignolas et al., 1995).

### Flavanone-3-Hydroxylase Expression Is Correlated With Taxifolin and Catechin Accumulation

In contrast to flavan-3-ol biosynthesis in Norway spruce, which was elucidated in previous work (Hammerbacher et al., 2014, 2018), very little is known about the synthesis of the dihydroflavonol taxifolin. We found a single high confidence transcript encoding a F3H enzyme in publically available gene sequence resources, and a single copy of this gene was also recorded in angiosperms such as petunia (Britsch et al., 1992), *Arabidopsis* (Pelletier and Shirley, 1996) and tea (Mahajan and Yadav, 2014). The existence of only a single copy of F3H in many plant species might be explained by the fact that other oxoglutarate-dependent dioxygenases in the flavonoid pathway such as flavonol synthase and anthocyanidin synthase can partially complement F3H activity (Buer et al., 2010).

In this study we showed that in Norway spruce *F3H* gene expression increased after infection with *E. polonica* and this correlated closely with taxifolin and catechin accumulation. On the other hand, in our transgenic lines in which *F3H* transcription was silenced by RNAi, lower levels of both metabolites were detected. This is in agreement with many other studies. For example, overexpression of F3H from plants such as *Lycium chinense* and tea in tobacco resulted in increased expression levels of late flavonoid biosynthesis genes and a significant increase in flavan-3-ols (Mahajan and Yadav, 2014; Song et al., 2016). *F3H* gene expression also correlates with plant developmental stages in which flavan-3-ols accumulate. This was shown in young and old tea leaves (Singh et al., 2008) as well as during heartwood development in black walnut (Beritognolo et al., 2002). All this evidence suggests that, like in other plant species, F3H plays an important role in the accumulation of not only taxifolin, but also down-stream metabolites in the flavonoid pathway of Norway spruce such as catechin and PAs.

### Taxifolin and Catechin Are Toxic to Both Bark Beetles and Their Fungal Associates

Accumulation of down-stream metabolites in the flavonoid pathway has often been shown to be a defense response to pathogen infection or herbivory. For example, the high accumulation of the flavan-3-ol catechin in poplar results in increased resistance to rust infection (Ullah et al., 2017). In this study we also demonstrated the *in vitro* anti-fungal activity of catechin against *E. polonica* at concentrations approximating the maximum amounts of compound produced by the saplings in this study. Interestingly, taxifolin was found to be even more toxic than catechin and a physiologically relevant mixture of catechin and taxifolin at the 2:1 ratio observed in pathogen-challenged cambium had an even greater effect than either of the compounds alone. Although taxifolin is known as a potent anti-oxidant (Weidmann, 2012) its function as an anti-microbial compound has not been fully elucidated yet. It may act by blocking the biotransformation or degradation of catechin. In previous work, the virulence of *E. polonica* was linked to its ability to degrade stilbene glucosides, flavan-3-ols such as catechin and procyanidin B1 and other spruce phenolics by oxidation, deglucosylation and by oxidative dimerization (Hammerbacher et al., 2013; Wadke et al., 2016). However, taxifolin was not a substrate for the fungal catechol dioxygenase enzymes involved in the oxidative cleavage of host phenolics and so might act as a competitive inhibitor (Wadke et al., 2016). Similarly, in studies on the grape pathogen *Botryotinia fuckeliana*, taxifolin-rhamnoside, and other phenolics were found to be strong inhibitors of the laccases (stilbene oxidases) that are responsible for oxidative stilbene dimerization (Goetz et al., 1999). The synergistic anti-fungal effect of the taxifolin-catechin mixture might therefore result from taxifolin-mediated inhibition of enzymes necessary for the detoxification of catechin. Synergy may also act at the level of sensory perception or by preventing compensatory herbivore feeding (Gershenzon et al., 2012).

Our results suggest that both catechin and taxifolin inhibit bark beetle tunneling and that the ingestion of these compounds had a visible effect on weight gain on this species. However, there were no differences in activity between catechin and taxifolin, and a mixture of the two compounds was no more toxic than the single compounds. The bioactivity of phenolics against insect herbivory is still debated (see Barbehenn and Constabel, 2011 for detailed review), but seems to depend on insect species. For example, catechin was identified as a feeding stimulant for willow leaf beetles (Doskotch et al., 1970; Kuokkanen et al., 2003) in contrast to other studies showing that this compound is a defense against herbivore feeding in oak trees (Feeny, 1970; Moctezuma et al., 2014). However, in the case of the spruce bark beetle, a previous study using a different bioassay set-up also showed that catechin and taxifolin inhibited tunneling behavior in *I. typographus* (Faccoli and Schlyter, 2007), supporting our hypothesis that high concentrations of these compounds could result in increased resistance to bark beetle attack in Norway spruce. Sensitivity to phenolics might also partially explain the close association of *I. typographus* with blue stain fungi known to degrade these compounds (Zhao et al., 2018).

Although our bioassays showed that both taxifolin and catechin play a role in tree defense against *I. typographus* and its associated fungi, other compounds might play a similar or even more important role in tree resistance. Concentrations of taxifolin-glucoside as well as PAB1 also increased significantly in response to fungal infection in this study. Experimental evidence is available showing that PAs have similar anti-fungal activity as catechin. For example, Ullah et al. (2017) showed that growth inhibition of the poplar rust fungus was equivalent when challenged with catechin and PAs. Similar results were also observed in growth trials of *E. polonica* with different concentrations of flavan-3-ols (Hammerbacher et al., 2014). However, the bioactivities of catechin and condensed tannins against insects are still not well understood and it is unknown if monomers are more active against insect feeding than polymers. It is also not known if taxifolin and its glucoside have similar modes of action. In a study by Auger et al. (1994) the biological activities of taxifolin and its glucoside were studied in pine against *Dipirion pini*, the common pine sawfly. The authors reported a toxic effect of both compounds on the sawfly but could not resolve the degree of toxicity of each. It is thought that glycosides are usually less toxic than the aglycone and that glycosylated compounds are activated during the plant defense response by deglycosylation (Morant et al., 2008). It was shown that *E. polonica* deglucosylates stilbenes (Hammerbacher et al., 2013). It is therefore possible that taxifolin glucoside can also be deglucosylated by the fungus, producing a more toxic compound that might impact negatively on its bark beetle vector. Clearly more research must be conducted to resolve the degree of toxicity of different flavonoids and other phenolic compounds in bark beetles and their associated fungi. Transgenic trees, such as the F3H RNAi lines produced in this study would be extremely valuable in addressing these questions, as *in vitro* experiments alone cannot reflect the full complexity of a living plant.

## CONCLUSION

In this study we identified two down-stream products of the flavonoid pathway in Norway spruce with activity against the bark beetle *I. typographus* as well as its fungal associate *E. polonica*. We also elucidated an unknown step in the Norway spruce flavonoid pathway for the biosynthesis of these compounds by characterizing a novel F3H enzyme. In plants, high F3H gene expression is not only known to increase resistance against biotrophic and necotrophic attackers (Mahajan and Yadav, 2014; Sun et al., 2016), but is also involved in increasing tolerance to cold, drought and heat stress (Mahajan and Yadav, 2014; Meng et al., 2015; Song et al., 2016). Selecting conifer genotypes with high F3H gene expression might therefore partially counteract the detrimental effects of climate change, as these genotypes might not only be more resistant to bark beetle attack, but also more resistant to abiotic stress which is the indirect trigger of large bark beetle outbreaks.

## Author Contributions

All authors wrote the manuscript. AH and JG funded this project out of grants received from the Max Planck Society and the University of Pretoria. AH, DK, AS, CU, and LPW performed the experiments and data analysis.

## Conflict of Interest Statement

The authors declare that the research was conducted in the absence of any commercial or financial relationships that could be construed as a potential conflict of interest.

## Footnotes

1 http://congenie.org/blast

2 http://mafft.cbrc.jp/alignment/server/

3 www.cambia.org

4 www.r-project.org

5 http://congenie.org
