# Supplementary material for: Flavanone-3-Hydroxylase Plays an Important Role in the Biosynthesis of Spruce Phenolic Defenses Against Bark Beetles and Their Fungal Associates
Source: Front Plant Sci. 2019 Feb 25;10:208. doi: 10.3389/fpls.2019.00208 (PMC6397876; doi:10.3389/fpls.2019.00208)
Supplement: Supplementary file 1 [file Table_1.docx]

**Supplemental Table 1:** NCBI-accession numbers of all the genes used for phylogenetic analysis of F3H. Numbers in bold are genes newly described in this study.

|  |  |
| --- | --- |
| **Species** | **GB Accession Number** |
| ***Picea abies*** | **MK256609** |
| ***Picea sitchensis*** | **MK256611** |
| ***Picea glauca*** | **MK256610** |
| *Pinus radiata* | AGY80772 |
| ***Pinus taeda*** | **MK256612** |
| *Ginkgo biloba* | AAU93347 |
| *Eucalyptus grandis* | XP_010033486 |
| *Pyrus communis* | AAX89399 |
| *Malus domestica* | AAX89397 |
| *Fragaria X ananassa* | AAU04792 |
| *Vitis vinifera* | NP_001268034 |
| *Theobroma cacao* | XP_007046698 |
| *Gossypum hirsitum* | XP_016735424 |
| *Petunia X hybrida* | AAC49929 |
| *Nicotinia tabacum* | NP_001312012 |
| *Populus trichocarpa* | ABK94757 |
| *Medicago truncatula* | AFK41061 |
| *Petroselinum crispum* | Q7XZQ7 |
| *Arabidopsis thaliana* | NP_190692 |
| *Brassica napus* | XP_022546782 |
| *Citrus sinensis* | NP_001275816 |
| *Citrus clementina* | XP_006425499 |
|  |  |

**Supplemental Table 2:** List of all the phenolic compounds analysed in the cambial region of the Norway spruce clone clone S21K0420232 7 days after wounding the stem with a cork borer. Mean concentrations of analytes were calculated from 5 biological replicates.

| *Analyte* | *Concentration (mg/g)* | *Standard Error (n=5)* |
| --- | --- | --- |
| **Stilbenes** |  |  |
| Astringin | 7.468 | 1.769 |
| Isorhapontin | 0.539 | 0.124 |
| Piceid | 0.079 | 0.010 |
| Piceatannol | nd | - |
| Rhapontigenin | nd | - |
| Resveratrol | nd | - |
|  |  |  |
| **Flavonoids** |  |  |
| Taxifolin glucoside | 1.563 | 0.312 |
| Catechin | 0.507 | 0.053 |
| Procyanidin B1 | 0.485 | 0.078 |
| Taxifolin | 0.305 | 0.038 |
| Naringenin | 0.028 | 0.006 |
| Eriodictyol | 0.027 | 0.005 |
| Gallocatechin | 0.007 | 0.003 |
| Catechin-gallocatechin dimers | nd | - |
| Quercetin glucoside | nd | - |
| Quercetin galactoside | nd | - |
| Quercetin | nd | - |
| Myricetin glucoside | nd | - |
| Myricetin | nd | - |
| Dihydromyricetin | nd | - |
|  |  |  |
| **Lignans** |  |  |
| Lariciresinol | 0.008 | 0.000 |
| Matairesinol | 0.001 | 0.000 |
| Nortrachelogenin | 0.007 | 0.002 |
| Pinoresinol | 0.008 | 0.001 |
| Secoisolariciresinol | 0.001 | 0.000 |
|  |  |  |

**Supplemental Table 3:** Accumulation of dihydrokaempferol and taxifolin after addition of 0.2 mg/mL of the respective substrates naringenin and eriodictyol to E. coli cultures transformed with PaF3H. The total culture volume was 105 ml.

|  |  |
| --- | --- |
| **Substrate** | **Product accumulation (mg/h)** |
| Naringenin | 0.332 ± 0.19 |
| Eriodictyol | 0.281 ± 0.13 |
|  |  |

**Supplemental Figures:**

**Supplemental Figure 1:** Alignment of the F3H open reading frames from *Picea abies, P. glauca, P. sitchensis and Pinus taeda.*

PaF3H atggcgcccgcagcagtggtggttagcgatcgacccgatttccttgtaacagcggaggag

PsF3H atggcgcccgcagcagtggtggttagcgatcgacccgatttccttgtaacagcggaggag

PgF3H atggcacccacatcagtcgtggtaagcgatcgaccggatttcctagtaacggcggaggta

PtF3H atggcgcccgcagcagtggtggctagcgatcgaccggatttccttgtaacagcagaggag

PaF3H ccgaagtccctgcaatccatcttcatccgagacgaggacgagcgccccaaggtggcttac

PsF3H ccgaagtccctgcaatccatcttcatccgagacgaggacgagcgccccaaggtggcttac

PgF3H ccgaagtccctgcagtccatcttcatccgagacgaggacgagcgccccaaggtagcttac

PtF3H ccgaagtccctgcagtccatcttcgtgcgagacgaggacgagcggcccaaggtggcttac

PaF3H aaccaattcagcaaagacattcccgtcatttccctttctggtattgaaggggcagagagg

PsF3H aaccaattcagcaaagacattcccgtcatttccctctctggtattgaaggggcagagagg

PgF3H aaccaattcagcaaagacattcccgtgatttccctctctggtatcgaaggggcagagagg

PtF3H aatcaattcagcaaagacattcccgtcatttccctctctggtatcgctggggaagaaagg

PaF3H ggccgagtgatagacgaggtgagcaaggcctgttctgaatggggaatattccaggtggtt

PsF3H ggccgagtgatagacgaggtgagcaaggcctgttctgaatggggattattccaggtggtt

PgF3H ggccgagtgatagacgaggtgagcaaggcctgttctgaatggggaatattccaggtggtt

PtF3H ggccgcgtgagagacgaggtgagcaaggcttgttctgaatggggaattttccaggtggtt

PaF3H gatcacggtgttcccaaggagctggtggattccatgactcggctctccagggacttcttc

PsF3H gatcacggtgttcccaaggagctggtggattccatgactcggctctccagggacttcttc

PgF3H gatcacggtgttcccaaggagctggtggattccatgactcggctctccaaggacttcttc

PtF3H gatcatggtgttccccaggaactggtggattccatgactcagctctccagggctttcttc

PaF3H gccctccctgcagaggagaagctcaaatacgacatgagaggtggagagcctggaggattt

PsF3H gctctccctgcagaggagaagctcaaatacgacatgagaggtggagagcctggaggattt

PgF3H gccctccccgcagaggagaagctcaaatacgacatgagaggtggaaagcgtggaggattt

PtF3H gcgctcccagcagaggagaagctcaaatacgacatgagaggtggaaagcgtggaggattt

PaF3H gtggtgagcagccatctccaaggcgaaagcgtcttcgactggagagagctctgcatttac

PsF3H gtggtgagcagccatctccaaggcgaaagcgtcttcgactggagagagctctgcatttac

PgF3H gtggtgagcagccatctccagggcgaaagcgtcctcgactggagagagatctgcacttac

PtF3H gtggtgagcagccatctgcagggcgaagccgttctggattggagagaaatctgcacttac

PaF3H ttctcatatccccttcaccagcgcgattacagtaggtggcctgtcaagcccgatggctgg

PsF3H ttctcatatccccttcaccagcgcgattacagtaggtggcctgtcaagcccgatggctgg

PgF3H ttctcctatccccttcaccagcgcgattacagtaggtggcctgtcaagcccgatggctgg

PtF3H ttctcttatccccttcaccagcgtgattacacaaggtggcctgtcaagcccgatggctgg

PaF3H agggagattgtggagaaatacagcgaagccctgatgggtttggcatgcaatatgttggcg

PsF3H agggagattgtggagaaatacagcgaagccctgatgggtttggcatgcaatatgttggcg

PgF3H agggagattgtggagaaatacagcgaagccctgatgggtttggcatgcaatatgttggcg

PtF3H agggacgttgtggagaaatacagcgaatcccttatgggtttggcatgcaagctgttgggg

PaF3H atagtatcagaggcattgggattggaatcggatgcagtgacgaaggcttgcgtggagatg

PsF3H atagtatcagaggcattgggattggaatcggatgcagtgacgaaggcttgcgtggagatg

PgF3H atagtatcagaggcattgggattggaatcggatgcagtgacgaaggcttgcgtggagatg

PtF3H ataatatcagaggcgttgggattggaaactgaagcagtgaccaatgcttgtgtggagatg

PaF3H gaccagaaagtggtggtaaacttctatccgaaatgcccagagccagacatgacgttggga

PsF3H gaccagaaagtggtggtaaacttctatccgaaatgcccagagccagacatgacgttggga

PgF3H gaccagaaagtggtggtaaacttctatccgaaatgcccagagccagacatgacgttggga

PtF3H gaccagaaagtggtgataaacttctatcccaaatgcccacaaccagacatgacactgggg

PaF3H ctgaagaggcacacagatccgggcaccatcactcttctcctccaagaccaagttggaggc

PsF3H ctgaagaggcacacagatccggtcaccatcactcttctcctccaagaccaagttggaggc

PgF3H ctgaagaggcacacagatccgggcaccatcactcttctcctccaagaccaagttggaggc

PtF3H ctgaaaaggcacaccgatccgggcaccatcactcttctcctgcaagaccaagttggaggg

PaF3H ctacaggctaccaaggacgatggcaaaaactggatcactgttgagcccattgaaggagcc

PsF3H ctacaggctaccaaggacgatggcaaaaactggatcactgttgagcccattgaaggagcc

PgF3H ctacaggctaccaaggacgatggcaaaaactggatcactgttgagcccattgaaggagcc

PtF3H ctacaagctactaaggacgatggaaaaaattggattactgtggagcccattcaaggagcc

PaF3H tttgttgtcaatttgggtgatcatatgcattatctgagcaatggcaagttcaagagcgcg

PsF3H tttgttgtcaatttgggtgatcatatgcattatctgagcaatgggaagttcaagagcgcg

PgF3H tttgttgtcaatttgggtgatcatatgcattatctgagcaatggcaagttcaagagcgcg

PtF3H tttgttgtcaatttgggtgatcatatgcattatctgagcaatggcaagttcaagagcgcg

PaF3H gaccatcaggcggtggtgaattcgaacagcagcaggctgtcgattgcgacctttcatagt

PsF3H gaccatcaggcggtggtgaattcgaacagcagcaggctgtcgattgcgacctttcataat

PgF3H gaccatcaggcggtggtgaattcgaacagcagcaggttgtcgattgcgacctttcagaat

PtF3H gaccatcaagcggtggtgaactcgaacagcagcaggctgtcgattgcgacgtttcagaat

PaF3H ccagcgccggagggcatcgtttacccgttggatggagttgtgaaggaggcggaaggcgag

PsF3H ccagcgccggagggaatcgtttacccgttggatggagttgtgaaggaggcggaaggcgag

PgF3H ccagcgcaggagggaatcgtgtacccgttggatggagttgtgaaggaggcggaaggcgag

PtF3H ccagcgcaggaggccatcgtgtacccgttggatggagtcgtgaaggaggcggaaggcgag

PaF3H aagtgcttcatggaagagcccatcacttacgctcagatgtactccagaaagatgagccta

PsF3H aagtgcttcatggaagagcccatcacttacgctcagatgtactccagaaagatgagccga

PgF3H aagtgcttcatggaagagcccatcactttcgctcagatgtacaccagaaagatgagccga

PtF3H aagtgcttcatggaagagcccatcacttttgctcagatgtactccagaaagatgagccga

PaF3H gacatagagctcgcccgccagaagaagatggccaaagttgctgagcaggaaaagtcgtga

PsF3H gacatcgagctcgcccgccagaagaagatggccaaagttgctgagcaggaaaagtcgtga

PgF3H gacatcgagctcgcccgccagaagaagatggccaaagttgctgagcaggaaaagtcgaga

PtF3H gacatcgagctcgctcgccagaagaagattgctaaacttcaagcgcaggagccggaagga

PaF3H ------------------

PsF3H ------------------

PgF3H gatcttccaatgatgtga

PtF3H g------caaaagcctaa


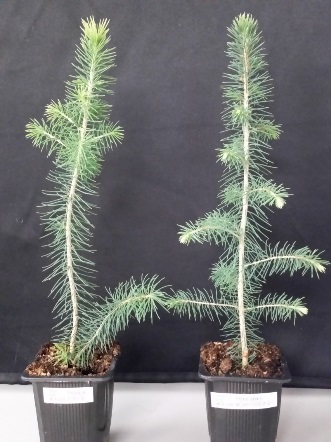


**Supplemental Figure 2:** Two-year old spruce samplings (left, PaF3H-RNAi line; right, vector-control) grown in 1 L pot.


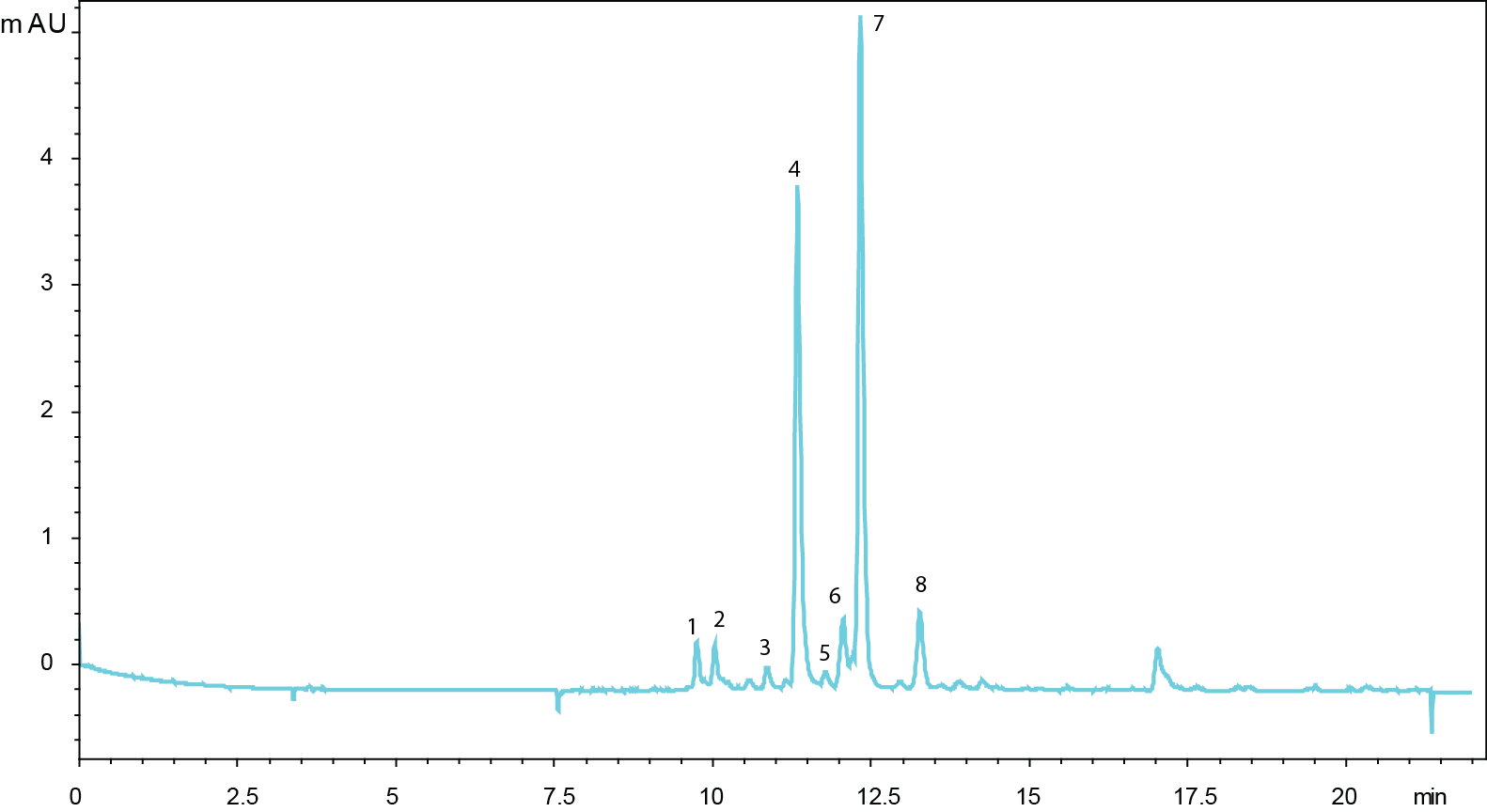


**Supplemental Figure 3:** Representative UV chromatogram (320 nm) of phenolics extracted from the bark and cambial region of a 40-year-old Norway spruce tree. (1) gallocatechin, (2)m/z 389, (3) catechin (4) *E-*astringin, (5) *Z-*astringin, (6) taxifolin-3-O-glucoside, (7) *E-*isorhapontin and piceid, (8) taxifolin
